# Using Barometer for Floor Assignation within Statistical Indoor Localization

**DOI:** 10.3390/s23010080

**Published:** 2022-12-22

**Authors:** Toni Fetzer, Frank Ebner, Frank Deinzer, Marcin Grzegorzek

**Affiliations:** 1 Faculty of Computer Science and Business Information Systems, University of Applied Sciences Würzburg-Schweinfurt, 97070 Würzburg, Germany; 2 Institute of Medical Informatics, University of Lübeck, 23562 Lübeck, Germany

**Keywords:** indoor positioning, sensor fusion, particle filter

## Abstract

This paper presents methods for floor assignation within an indoor localization system. We integrate the barometer of the phone as an additional sensor to detect floor changes. In contrast to state-of-the-art methods, our statistical model uses a discrete state variable as floor information, instead of a continuous one. Due to the inconsistency of the barometric sensor data, our approach is based on relative pressure readings. All we need beforehand is the ceiling height including the ceiling’s thickness. Further, we discuss several variations of our method depending on the deployment scenario. Since a barometer alone is not able to detect the position of a pedestrian, we additionally incorporate Wi-Fi, iBeacons, Step and Turn Detection statistically in our experiments. This enables a realistic evaluation of our methods for floor assignation. The experimental results show that the usage of a barometer within 3D indoor localization systems can be highly recommended. In nearly all test cases, our approach improves the positioning accuracy while also keeping the update rates low.

## 1. Introduction

Computer-controlled localization has been a subject of interest in computer science over the past two decades. Especially in the upcoming world of smart things, such as modern cars, Industry 4.0, or even smart homes, position awareness is a central problem. Considering the self-localization of a human, GPS (*Global Positioning System*) has made its way into almost all mobile devices. However, for indoor environments, the GPS signal is attenuated by dense, sometimes reflective, construction materials such as walls or ceilings, that interrupt the line of sight to the satellites [1]. This leads to high errors due to the significant loss of signal strength and multipath propagation, resulting in an accuracy of tens of meters. Such accuracy is not sufficient for most scenarios inside buildings, which is why many alternative methods have been developed in recent years [2,3,4]. One of the most important tools for estimating the possible location of a pedestrian in a building is sensor fusion [5,6,7].

In recent years, a variety of sensors, e.g., Wi-Fi, magnetometer, or camera, have been investigated for indoor localization applications [8]. Among them, one of the newest and least known is the barometer. Especially, expensive smartphones have been equipped with barometric sensors [9]. It measures the atmospheric pressure, and since the pressure varies mainly with altitude, it is well suited for detecting floor changes. To account for measurement errors and noise, we use a probabilistic approach that models the uncertainties of the sensors as a probability density function. Such a probabilistic approach allows for a unified interface for the purpose of sensor fusion, e.g., the combination of different sensor types for state estimation [10].

Obviously, atmospheric pressure is the pressure within the atmosphere of the earth. In a nutshell, it is generated by the weight of the air column standing on the earth’s surface or on an object. Therefore, as altitude increases, atmospheric pressure decreases accordingly. Different environmental factors, such as temperature, weather patterns, and humidity, have an impact on this relationship. In addition to these environmental factors, the MEMS barometers built into smartphones are noisy and sensitive to rapid changes. The authors of [11] have examined different elevation scenarios and found that even opening a window or a door can significantly alter pressure readings. Further, different air conditioning characteristics between two adjacent rooms can cause such a behavior. They conclude that while pressure measurements cannot be directly converted into accurate vertical information, such as altitude, in some cases, low-cost MEMS barometers provide very useful information for localization applications.

**Figure 1:**
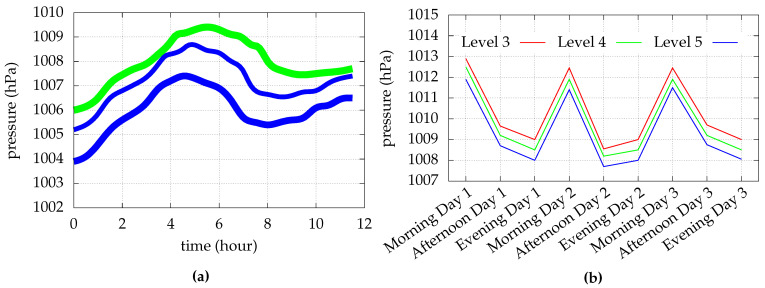
Recordings of absolute pressure readings for two scenarios. (**a**) 12 h pressure trend on a single floor using a Galaxy S3 (green) and two Nexus 4 (blue). The width of the lines describes the uncertainty of the measurements. (**b**) Recordings of three pressure readings on three different levels at three different times across three different days. The figures are based on the datasets provided by [9].

In Figure 1a, a 12 h pressure trend on a single floor using a Galaxy S3 (green), having an STM LPS331AP MEMS pressure sensor [12], and two Nexus 4 (blue), equipped with a Bosch BMP 180 MEMS pressure sensor [13], is illustrated. The recordings are based on the datasets provided by [9]. For the duration of the recording, all devices lay flat on a table in a closed room. It can be seen that not only the time of day and thus a temperature variation influences the pressure readings, but also the different devices used. This causes an average pressure difference of 1.2 hPa (hectopascal), or ∼10m of difference in height, between the two Nexus devices.

The behavior of atmospheric pressure readings at various floor levels and on different days is further explained in Figure 1b. It can be seen that pressure measurements exhibit considerable diurnal variants, yet the difference in pressure between different floor pairs is remarkably constant and continuous. These findings demonstrate that absolute pressure measurements are challenging to use for floor identification, even with reference measurements, when two identically constructed devices produce different readings. Therefore, this work uses the relative difference between the barometer pressure readings over time.

Nevertheless, there are many approaches in the literature that use absolute pressure measurements to determine a floor. For example, the authors of [14] use reference devices on each floor. By comparing the reference and the pressure measured on the smartphone, the floor can be determined. In order to eliminate the deviation between all involved barometric sensors, a calibration of the to-be-localized smartphones as well as the reference devices must be carried out. Here, environmental influences can be taken into account via meteorological information, for example from a nearby weather station [15]. However, for large and complex buildings, this approach may fail if the number of reference devices is too small. In addition, the calibration of all devices involved is complex and must be corrected even for small changes, such as increased humidity. This requires additional sensors per reference device for a very accurate calibration.

The authors of [16] utilize a crowd-based fingerprinting approach. Their goal is to identify the floor level the user is currently in without absolute knowledge. For this, a fingerprinting map is constantly updated by everyone using the system. At first, a calibration for normalizing the barometer readings between different devices carried by users is conducted. Based on this, the pressure measurements are then clustered, allowing for the fingerprinting approach. Consequently, the number of resulting clusters should then be identical to the number of floors. While this approach provides good results, updating the fingerprinting map requires that people are continuously present in the building and have the corresponding application installed. For many practical applications, however, such an approach is not a viable option.

In this work, a straightforward approach is taken that imposes few conditions and prior knowledge on the system. We do not identify the exact floor number by using some kind of reference or crowed-based system. The presented method just recognizes if a floor was changed. All we need beforehand is the ceiling height including the ceiling’s thickness. Considering a sensor fusion, additional sensors will reduce the uncertainty of being in another floor. In contrast to state-of-the-art methods, our statistical model uses a discrete state variable as floor information, instead of a continuous one. Finally, by using environmental information and a state transition to model the pedestrian’s movement, we are able to determine time and position of a floor change.

The paper is organized as follows. Section 2 gives an overview of the recursive state estimation. The basics of our indoor localization approach including the used state transition are shown in Section 3. Section 4 presents the statistical floor assignation approach and theoretical thoughts. Section 5 shows the experimental results, while Section 6 provides the conclusion of this work and prospects of future work.

## 2. Recursive State Estimation

In indoor localization, we are interested in finding the current location of a pedestrian inside a building using a series of observations ${\langle o\rangle }_{t}=o_{1},o_{2},\ldots ,o_{t}$ provided at time *t* by a multitude of sensors. Such a system suffers from uncertainties due to the imperfection and incompleteness of the sensors and used methods. Therefore, we treat the localization problem with a probabilistic representation to draw inferences about a hidden state of the system $q_{t}$. A broad class of methods that satisfies the Markov property are Bayes filters, which provide a recursive state estimation by inferring the probability density

(1) $$p\left(q_{t}|{\langle o\rangle }_{t}\right)=\underset{\mathrm{evaluation}}{\underset{︸}{p(o_{t}|q_{t})}}\int \underset{\mathrm{transition}}{\underset{︸}{p(q_{t}|q_{t-1})}}\underset{\mathrm{recursion}}{\underset{︸}{p(q_{t-1}|{\langle o\rangle }_{t-1}}})dq_{t-1}.$$

The estimation of the hidden state is then performed using the two steps state transition $p(q_{t}|q_{t-1})$ and state evaluation $p(o_{t}|q_{t})$. The recursion starts with an initial (uniform) distribution $p(q_{0})$ and contains all information up to time *t*.

In the localization system used in this work, as presented in our previous works, the state $q_{t-1}$ will be integrated in the evaluation; our probability density can be rewritten similar to [17] as:

(2) $$p\left(q_{t}|{\langle o\rangle }_{t}\right)=p\left(o_{t}|q_{t},q_{t-1}\right)\int p\left(q_{t}|q_{t-1}\right)p\left(q_{t-1}|{\langle o\rangle }_{t-1}\right)dq_{t-1}\;.$$

Finding analytical solutions for (1) or (2) is only possible in rare cases or requires for further assumption [5]. We therefore used sequential Monte Carlo, namely the CONDENSATION particle filter, as an approximation method [18]. The filter behaves identically to the better known Bootstrap particle filter by using the state transition as proposal distribution and weights a set of sample (particles), which are used to approximate the posterior, based on the evaluation density [19]. In addition, a resampling step is utilized to handle the phenomenon of weight degeneracy [20].

## 3. Statistical Indoor Localization

As mentioned above, our novel approach recognizes if a floor was changed without any initial positioning information. Considering an indoor localization system, we need to incorporate other sensor models to determine the time and position of a floor change. By assuming a statistical independence between all sensors and their models, we can easily describe the sensor fusion procedure as

(3) $$p\left(o_{t}|q_{t},q_{t-1}\right)=\prod_{i=1}p{\left(o_{t}|q_{t},q_{t-1}\right)}_{i}.$$

Here, every component *i* refers to a probabilistic sensor model, providing a weight to the samples of the particle filter. For example a combination of a sensor model for Wi-Fi and an activity recognition is presented in [7] or the utilization of Wi-Fi FTM for absolute positioning information and a pedestrian dead reckoning (PDR) in [21].

For describing the *likelihood* in (2) of observing a sensor measurement $o_{t}$, the state $q_{t}$ is given by

(4) $$q_{t}={\left(x_{t},y_{t},z_{t},\alpha_{t},\rho_{t}\right)}^{T}\;,x_{t},y_{t},\alpha_{t},\rho_{t}\in R,\;z_{t}\in N.$$

It represents a pedestrian’s possible whereabouts, e.g., location and orientation. The position is represented by $x_{t},y_{t},z_{t}$, where $z_{t}$ is discrete. This results in a mixed discrete/continuous state space [22]. The heading is given by $\alpha_{t}$ and $\rho_{t}$ provides the atmospheric pressure in hectopascal (hPa) at time *t*.

According to the CONDENSATION algorithm, the state transition model $p(q_{t}|q_{t-1})$ is used to draw new states at every filter update. For this, we use a graph-based transition model instead of some simple density function. The method was first introduced in our previous work [23]. Based on the building’s floorplan, this model only allows for walks that are actually feasible. The floorplan is described by an equally spaced grid of s=20cm squares for each of the building’s floors. To account for walls and other obstructions, only areas of the floorplan that do not intersect with such are designated as squares and are thus actually walkable. Each square is represented as vertex $v_{x,y,z}\in V$ denoting its center within a graph G=(V,E). A new state $q_{t+1}$ is then randomly drawn from all valid moves of a state $q_{t}$ on G. This is also known as random graph walks [24].

The undirected edges *E* of the graph G are described by

(5) $$e_{v_{x+i,y+j,z}}^{v_{x,y,z}}\in E,\;i,j\in \left\{-1,0,1\right\},\;i\neq 0\vee j\neq 0,$$

connecting all vertices with their immediate neighbors in the x,y-plane, if such a neighbor exists. Edges $e_{v_{x,y,z^{\prime }}}^{v_{x,y,z}}$ within the *z*-plane are added for all (x,y) within regions of staircases or elevators. New states $q_{t+1}$ may now be sampled by:

(1) Drawing a distance *d* from a distribution that, depending on the interval between successive transitions, resembles the gait and speed of a pedestrian.
(2) Obtaining the vertex $v_{x,y,z}$ in which the old state $q_{t}$ resides.
(3) Walking randomly along adjacent edges depending on their probability $p(e|q_{t})$ and subtracting their length from *d* until d≤0 is reached
(4) Slightly scattering the final destination by picking a random position within the square. The target vertex $v_{x^{\prime },y^{\prime },z^{\prime }}^{\prime }$ denotes:
  (6) $$\left(x_{e},y_{e},z\right),\;x_{e}∼U\left(x^{\prime }\pm \frac{s}{2}\right),\;y_{e}∼U\left(y^{\prime }\pm \frac{s}{2}\right).$$

The pedestrian’s walking behavior is modeled by $p(e|q_{t})$. As we assume them to walk almost straight, each edge’s probability depends on the currently estimated heading $\alpha_{t}$:

(7) $$p\left(e|q_{t}\right)=N\left(\Delta \alpha_{t}|0,\sigma_{\mathrm{dir}}^{2}\right),\;\Delta \alpha_{t}=\alpha_{t}-∠e.$$

$\sigma_{\mathrm{dir}}$ is a small value of about 5${}^{\circ }$, limiting the likelihood of deviating from walking a straight line. Depending on the edge $e^{\prime }$ drawn according to this probability, we update the estimated heading $\alpha_{t}=∠e^{\prime }$ before drawing the next edge until d≤0. This ensures a mainly straight walking behavior while still allowing for turns and bends.

## 4. Floor Assignation

Considering a running statistical indoor localization, the first step of our floor assignation approach is to model a new state $q{}_{t}$. The switch of a state $q{}_{t}$ between two floors and the fitting of the relative altitude $\rho_{t}$ is performed within our state transition $p(q_{t}|q_{t-1})$ presented in the previous chapter. As mentioned before, movement is only allowed on an edge of the floor graph. The same applies for a change of the floor level. We predict $\rho_{t}$ using

(8) $$\rho_{t}+=\mathrm{sgn}\left(z_{t-1}-z_{t}\right)\cdot N\left(\rho_{z},\sigma_{\mathrm{baro}}^{2}\right),$$

where $\rho_{z}$ is the change of pressure between the past and current floor. We consider the variation of pressure by drawing randomly from a normal distribution. The uncertainty of the used barometer is described by $\sigma_{\mathrm{baro}}$.

There are several possible methods for calculating $\rho_{z}$. If environmental information is provided the change of pressure between two floors can be calculated using the barometric formula [25]:

(9) $$\rho \left(h_{1}\right)=\rho \left(h_{0}\right){\left(1-\frac{a\Delta h}{T(h_{0})}\right)}^{\frac{Mg}{Ra}}$$

The universal gas constant *R* is fixed and for most cases also the molar mass *M* and the gravitational acceleration *g* can be assumed to be constant. For the sake of simplicity, we assume a constant lapse rate *a*, temperature $T(h_{0})$ are the same for the whole building and do not change in the current run. Both values can of course be easily determined by using a nearby weather station for *a* and the current room temperature with a simple thermostat for $T(h_{0})$. Updating them frequently would increase the stability of (9) and thus would compensate for rapid pressure changes as discussed in the introduction of this work. Here, $h_{1}$ and $h_{0}$ represent the elevation (height above sea level) of a single floor. The change of pressure $\rho_{z}$ would then be calculated by

(10) $$\rho_{z}\approx \left|\rho \left(h_{1}\right)-\rho \left(h_{0}\right)\right|.$$

If no information about the exact elevation $h_{0}$ of the building is given, a GPS-based value or the mean sea level pressure (MSLP) could be used instead. This would affect the accuracy again, but has the advantage that all we need beforehand is the ceiling height including the ceiling’s thickness represented by Δh. Another possible way to obtain $\rho_{z}$, is by simply measuring the change in pressure with a reference device. Referring to the weaknesses shown in Section 1, this could result in a difficult and complex task. If preparation time is short and every floor has the same height, a constant value $\rho_{\mathrm{const}}$ using

(11) $$\rho_{z}=\left(z_{t-1}-z_{t}\right)\cdot \rho_{\mathrm{const}}$$

could also be considered.

After the transition step is performed, the state is evaluated using an observation $o_{t}$. At every time step *t*, a measurement $\rho_{{\mathrm{baro}}_{t}}$ of the barometer is given in hectopascal. Nearly all consumer devices provide the atmospheric pressure above sea level. We evaluate each possible state $q{}_{t}$ as follows:

(12) $$p{\left(o_{t}|q_{t}\right)}_{\mathrm{baro}}=N\left(\rho_{t}|\rho_{{\mathrm{rel}}_{t}},\sigma_{z}^{2}\right).$$

where $\rho_{{\mathrm{rel}}_{t}}$ denotes the relative altitude difference with regard to some reference measurement $\rho_{\mathrm{ref}}$. $\rho_{t}$ is again the relative altitude of a state $q{}_{t}$. We assume a normal distributed error and thus chosen a Gaussian as density function for $p{\left(o_{t}|q_{t}\right)}_{\mathrm{baro}}$. The relative uncertainty of being in the current floor is thus described by the standard deviation $\sigma_{z}=\frac{\rho_{z}}{2}$.

Our approach provides two possibilities for determining $\rho_{{\mathrm{rel}}_{t}}$: the reference measurement $\rho_{\mathrm{ref}}$ is recorded at the beginning of the localization process or using a size-limited measurement history. The first case is pretty easy and straight forward with

(13) $$\rho_{{\mathrm{rel}}_{t}}=\rho_{{\mathrm{baro}}_{t}}-\rho_{\mathrm{ref}},$$

where $\rho_{\mathrm{ref}}=\rho_{{\mathrm{baro}}_{0}}$. In the second case, we are also using (13), but $\rho_{\mathrm{ref}}$ is determined periodically. For periodical determination a circular buffer [26] with fixed memory size (e.g., 30 s) can be used, where $\rho_{\mathrm{ref}}$ is the oldest entry. The size depends on the average time steps *t* needed for changing a floor. It should be noted, that a walk on the same floor level, longer then the current memory size, would result in $\rho_{\mathrm{ref}}=0$. Therefore, we have to change the state transition step in (8) as follows:

(14) $$\rho_{t}=\left(z_{t-1}-z_{t}\right)\cdot N\left(\rho_{z},\sigma_{\mathrm{baro}}^{2}\right),$$

The benefit of using a small history size is to counteract environmental changes on long runs. In order to increase the stability of $\rho_{{\mathrm{rel}}_{t}}$ a simple low-pass filter such as

(15) $$\rho_{{\mathrm{rel}}_{t}}=\rho_{{\mathrm{rel}}_{t-1}}+\alpha \cdot \left(\rho_{{\mathrm{rel}}_{t}}-\rho_{{\mathrm{rel}}_{t-1}}\right)$$

could be used for smoothing purposes.

## 5. Experimental Results

The experiments were carried out in a building of the University of Applied Sciences Würzburg-Schweinfurt. We included all floors (0 to 3), each about 77m×55m in size. Recordings were collected by five different subjects on two testing paths using a Google Nexus 5 and a Samsung Galaxy S5 at the same time. The first path is 120m meters and takes three minutes to complete, while the second is 220m and takes five minutes. A path is given by markers located on the ground. Whenever a test person passes one of these markers, he or she taps a button in the recording app, which saves the current timestamp. A constant movement speed is assumed between two consecutive markers. The position error can then be calculated by comparing the current position estimate with the ground truth position interpolated at that time. This procedure does of course not provide a perfectly accurate ground truth, but still remains within the error tolerance. If a test person forgot to save the timestamp, the run had to be repeated because no timestamp could be assigned to the marker in question. The computation was performed offline using 10,000 particles for approximation. The position estimation is calculated using the weighted arithmetic mean over all particles for every filter update at time *t* [27].

Besides the barometer, we incorporated the following sensor models presented in our previous works: Wi-Fi, iBeacons, step and turn detection. The fusion of the sensors is performed using (3) as follows:

(16) $$\begin{array}{cc} p(o_{t}|q_{t},q_{t-1})= & p{\left(o_{t}|q_{t},q_{t-1}\right)}_{\mathrm{turn}}p{\left(o_{t}|q_{t},q_{t-1}\right)}_{\mathrm{step}}\\ \; & p{\left(o_{t}|q_{t}\right)}_{\mathrm{baro}}p{\left(o_{t}|q_{t}\right)}_{\mathrm{ibeacon}}p{\left(o_{t}|q_{t}\right)}_{\mathrm{wifi}}. \end{array}$$

The Wi-Fi model $p{\left(o_{t}|q_{t}\right)}_{\mathrm{wifi}}$ and the iBeacon model are using a multilateration based on received signal strength indications (RSSI) [20,28]. A probability is obtained by comparing RSSI measurements with an RSSI estimation. The RSSI estimate between the transmitter and the potential position is obtained using a radio propagation model, such as the log-distance model. On each floor there are five commercial Wi-Fi access points (access-points) installed. Furthermore, we use six iBeacons in the respective staircases for stabilization, as Wi-Fi is strongly shielded there by the reinforced concrete walls. Unfortunately, for legal and security reasons, we are not allowed to add the positions of the Wi-Fi access-points to the figures or provide further detailed information about them. The model parameters for Wi-Fi and iBeacons were $P_{0_{\mathrm{wifi}}}=-40\;\mathrm{dBm}$, $\gamma_{\mathrm{wifi}}=2.7$ and $\gamma_{\mathrm{ibeacon}}=1.9$. $P_{0_{\mathrm{ibeacon}}}$ was set according to each iBeacon’s configuration. For uncertainties we used $\sigma_{\mathrm{wifi}}=\sigma_{\mathrm{ibeacon}}=8.0$ both growing with each measurement’s age. The models for step and turn detection are described in [23]. To briefly explain, both models compare the measured values, i.e., distance and heading, with the trajectory of a sample between time *t* and t−1 resulting from the transition step. The step evaluation uses the parameters $d_{\mathrm{step}}=50\;\mathrm{cm},\sigma_{\mathrm{step}}=10\;\mathrm{cm}$ if a step was recognized and $d_{\mathrm{nostep}}=0\;\mathrm{cm},\sigma_{\mathrm{nostep}}=5\;\mathrm{cm}$ if not. To evaluate the heading, we use $\sigma_{\mathrm{heading}}=50\;{}^{\circ }$.

**Figure 2:**
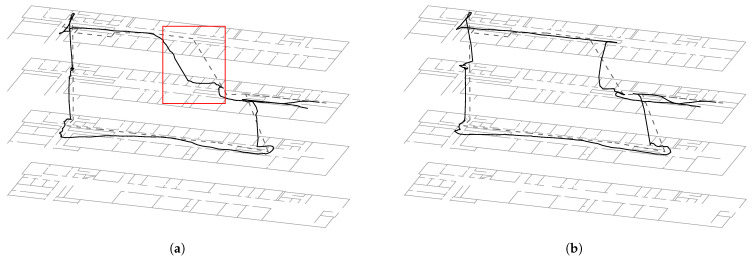
A typical localization results for the first path using (**a**) all sensors except the barometer model and (**b**) including it. The path is 220m long and it takes 5min to walk it.

Figure 2a shows a typical localization result for the first path using all sensors except the barometer. The walk starts on the right side of the second floor, walking up to the third floor and so on. The ground truth path is marked as a dotted line. By entering the first floor on the left-hand side of the picture, we rotated the phone into landscape mode. This is the reason for the prolonged curve at this position. The update time is set on 500ms, since it is approximately the time a person need to make one step. The height of the 1st, 2nd, and 3rd floor is 3.4m including the ceiling’s thickness. It can be clearly seen that the first floor change (cf. red square) was detected quite late. This can be explained by the poor Wi-Fi coverage in this area. The average of the error between ground truth and approximation is ${\bar{x}}_{\mathrm{noBaro}}=345\;\mathrm{cm}$ with a standard deviation of 193cm.

By incorporating the barometer, we are now able to recognize the correct position for this floor change. This can be seen in Figure 2b. Here, we determined $\rho_{z}$ using the barometric formula, where $\rho (h_{0})$ was set to the MSLP and $\sigma_{\mathrm{baro}}=0.005$. The reference value $\rho_{\mathrm{ref}}$ was recorded at the beginning of the walk. Due to the smoothing behavior of our approach, the approximated path was slightly straightened. Now the average has improved to ${\bar{x}}_{\mathrm{Baro}}=312\;\mathrm{cm}$ with a standard deviation of 219cm. Comparing Figure 2a,b, it is only logical that no huge improvement could be expected, but nevertheless a better overall approximation. Since all visited floors are the same height, a constant value $\rho_{z}=0.3$ or a size-limited measurement history for $\rho_{\mathrm{ref}}$ supply similar results.

**Figure 3:**
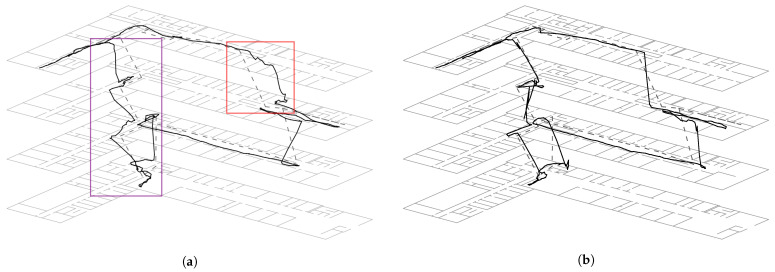
A typical localization results for the second path using (**a**) all sensors except the barometer model and (**b**) including it. The path is 120m long and it takes 3min to walk it.

Another example of our approach can be seen in Figure 3. Here, we are using the same setup as above. The route starts on the left side of the third floor. Walking alongside the same floor until the starting point of the first path, which is on the right side of the building in the second floor. After doing a $180\;{}^{\circ }$ turn, we walk down to the first and then to the zeroth floor. The route ends by walking the zig-zag staircases back to the start. Again, a visible improvement of the floor changes and also the position awareness can be seen by comparing Figure 3a,b. However, looking at the error between ground truth and approximation the localization result without barometer provides ${\bar{x}}_{\mathrm{noBaro}}=315\;\mathrm{cm}$ with a standard deviation of 222cm, whereas ${\bar{x}}_{\mathrm{Baro}}=323\;\mathrm{cm}$ and 283cm. This is a result of a series of timing errors during the long stair descent at the end of the second path.

One reason for the timing errors is the non-optimal ground truth model. For example, as shown in Figure 2, the ground truth path (dashed line) runs in a vertical straight line from floor 3 directly to floor 1, but it obviously does not model the architecture of the staircase. This actually consists of several zigzag staircases, each with a level between two floors and on each floor. As described in Section 3, the transition draws particles based on a constant walking speed between the floors and thus behaves identically to the ground truth model. In contrast, when including the barometer evaluation model each sample is weighted based on real measurements, which is directly proportional to the vertical movement speed of the subject. Since the speed of movement on a staircase is very different from the speed of movement on a plane surface, as assumed for the movement on the graph, the above mentioned timing problems occur. Assuming a more realistic ground truth model, the localization with barometer should lead to a lower approximation error or at least not worsen it further.

The above discussion reveals a more general problem of the approach presented here, namely the discrete representation of the floors. Moving between floors on a discrete basis essentially ignores any stairs and is therefore more like jumping between them. It is therefore also not possible to model positions between two floors. If a user is in the center of a stairwell, the measurement of the atmospheric pressure may confirm this, but the transition is not able to model the density in this area, which is why an incorrect approximation of the posterior results even after weighting with the evaluation model. Put more simply, the particle filter does not have samples on the stairs that are weighted according to the barometer readings and thus cannot provide a suitable approximation of the state-space model.

Another problem was noticed in the experiments, which can be traced back to the discrete floor changes. When changing floors, the pressure changes proportionally to the vertical height and is therefore directly dependent on the walking speed of the test person. During the first half of a staircase, the evaluation therefore ensures that samples in the floor that has just been left are still rated higher than in the one to be entered. However, the step detection as well as the transition continue for every filter update and causes the entire density to shift in the direction of the specified heading. Especially with high floors or if other sensor models such as Wi-Fi also consider the previous floor as more likely, this can lead to an under-representation of samples of the particle filter on the new floor. Ultimately, this leads to the fact that all samples of the particle filter are past the stair, so that no transition between floors is possible anymore and thus ultimately the floor change has failed. This problem is also known as sample impoverishment [20].

In contrast to the other, the zeroth floor is 70cm higher. Nevertheless using a constant $p_{z}$ or a size-limited measurement history for $\rho_{\mathrm{ref}}$ results again in a similar position estimation. This is no surprise since we have chosen a very wide $\sigma_{z}^{2}$ in (12) due to the fluctuating measurements of the phone’s barometric sensor and thus 70cm is not a great deal. Considering even higher floors these methods are very likely doomed to failure.

## 6. Conclusions and Future Work

We successfully presented the integration of the phone’s barometer within an indoor localization system. Our method is able to detect floor changes and thus increase the accuracy of the estimated position. It could be seen that absolute pressure readings are highly affected by different influences such as temperature, weather patterns, or the used hardware, which confirms our relative approach. By using a recursive state estimation an unified interface for incorporating highly different sensor types is also given. To enable a realistic evaluation, we additionally incorporate Wi-Fi, iBeacons, Step, and Turn Detection statistically. It could be seen that the barometer is a very good addition to those sensors. Especially, the timing of floor changes as well as the approximated path has improved visibly. In addition, the very limited method for floor estimation using a constant change of pressure for every floor provides similar results. The same applies for the size-limited measurement history. The usage of both methods still depends heavily on the area of application. At this point in time, the presented approach only allows discrete floor changes, i.e., de facto jumps over several meters. However, extending this to a continuous representation for the map, for example a navigation mesh, should be straightforward, but requires more precise specifications for the barometric formula as previously discussed. An additional use of the accelerometer to detect vertical movements could stabilize this approach and possibly even remove the requirement that the heights of the floors must be known. Statistically integrating the detection of the transportation mode, namely elevator, escalator, or stairs, is another prospect of future work. This would provide additional position awareness.

## Data Availability

Not applicable.
